# Analysis of hydroxychloroquine prescriptions for COVID‐19 in Japan

**DOI:** 10.1002/jgf2.592

**Published:** 2023-02-27

**Authors:** Takashi Watari, Yasuharu Tokuda, Hiromizu Takahashi, Kiyosu Taniguchi, Kenji Shibuya

**Affiliations:** ^1^ General Medicine Center Shimane University Shimane Japan; ^2^ Department of Medicine University of Michigan Medical School Ann Arbor Michigan USA; ^3^ Muribushi Okinawa Clinical Training Center Okinawa Japan; ^4^ Tokyo Foundation for Policy Research Tokyo Japan; ^5^ Department of General Medicine Juntendo University Faculty of Medicine Tokyo Japan; ^6^ National Hospital Organization Mie National Hospital Mie Japan

## Abstract

Number of hydroxychloroquine prescriptions per month for patients with coronavirus disease 2019 (COVID‐19) in Japan from January 2020 to November 2021. The blue bars show the monthly number of chloroquine prescriptions for COVID‐19 among patients in the Medical Data Vision database, which includes data on approximately 20% of acute care hospitals in Japan. The gray line shows the national number of COVID‐19 notifications in Japan by month over the same period.

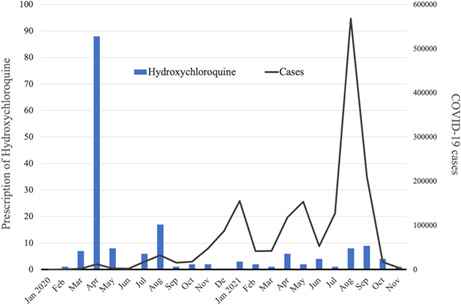

## FUNDING INFORMATION

This research did not receive any specific grant from funding agencies in the public, commercial, or not‐for‐profit sectors.

## CONFLICT OF INTEREST

None declared.

## ETHICS STATEMENT

This study was approved by the institutional ethics committee of the Muribushi Okinawa Center for Teaching Hospitals.

To the Editor,

Hydroxychloroquine was once considered a candidate for treating coronavirus disease 2019 (COVID‐19)^1^, ^2^ but is no longer recommended. In March 2020, the former US President Donald Trump recommended it as “one of the biggest game‐changers in medical history and needs to be used immediately.”^3^ On March 28, 2020, the United States Food and Drug Administration (US FDA) approved hydroxychloroquine for emergency use for COVID‐19.^3^ We assessed the trend in the monthly number of hydroxychloroquine prescriptions for patients with COVID‐19 in Japan from January 16, 2020, to November 30, 2021, using a hospital‐based administrative database (Medical Data Vision, Tokyo, Japan), to determine the effect of mass media publicity on the prescribing patterns of physicians in Japan. The Medical Data Vision database includes data on 32 million patients and covers over 350 healthcare institutions (20% of acute care hospitals) in Japan. This study was approved by our hospital's institutional ethics committee.

**FIGURE 1:**
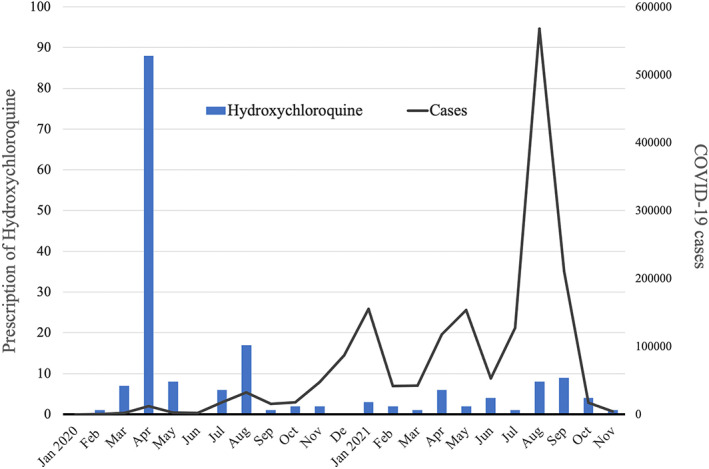
Number of hydroxychloroquine prescriptions per month for patients with coronavirus disease 2019 (COVID‐19) in Japan from January 2020 to November 2021. The blue bars show the monthly number of chloroquine prescriptions for COVID‐19 among patients in the Medical Data Vision database, which includes data on approximately 20% of acute care hospitals in Japan. The gray line shows the national number of COVID‐19 notifications in Japan by month over the same period.

Of 140,451 COVID‐19 patients, 173 (0.12%) were treated with hydroxychloroquine. The number of hydroxychloroquine prescriptions peaked in April 2020, during the first wave (88 prescriptions) and decreased to less than 10 per month after September 2020, despite an increasing number of COVID‐19 cases being reported nationwide during subsequent waves of the pandemic (Figure 1). Hydroxychloroquine prescription did not differ by patients’ gender, but it was more likely to be prescribed to older patients than to younger patients.

Although the number of hydroxychloroquine prescriptions issued in Japan was considerably lower than that in the United States (US), the secular trend in Japan followed that in the United States, where the number of prescriptions increased from 1143 in February 2020 to 75,569 in March 2020 and continued to increase for several months.^2^, ^4^ Although the Japanese government did not recommend hydroxychloroquine,^2^, ^5^ Japanese physicians might have been influenced by Japanese media reports that hydroxychloroquine was a potentially promising treatment for COVID‐19. After the US FDA declared an emergency revocation of its use permit on June 15, 2020, due to lack of evidence of efficacy in randomized controlled trials and concern about cardiotoxicity, very few prescriptions were issued in Japan.^3^, ^4^, ^5^

Although the data do not represent all prescriptions of hydroxychloroquine for COVID‐19 in Japan, they are likely to be representative of the Japanese population. This analysis has some limitations. We did not have data on the specialties of the physicians who prescribed hydroxychloroquine and were unable to assess whether hydroxychloroquine was prescribed in response to patient requests.

These results suggest that the prescription patterns in Japan might have been influenced by media reports, as in the United States.

## Data Availability

The data that support the findings of this study are available from the corresponding author, T.W., upon reasonable request.
